# High BMI levels associate with reduced mRNA expression of *IL10* and increased mRNA expression of *iNOS* (NOS2) in human frontal cortex

**DOI:** 10.1038/tp.2016.259

**Published:** 2017-02-28

**Authors:** J K Lauridsen, R H Olesen, J Vendelbo, T M Hyde, J E Kleinman, B M Bibby, B Brock, J Rungby, A Larsen

**Affiliations:** 1 Department of Biomedicine, Aarhus University, Aarhus C, Denmark; 2 Lieber Institute for Brain Development, Baltimore, MD, USA; 3 Department of Psychiatry, Johns Hopkins School of Medicine, Baltimore, MD, USA; 4 Department of Neurology, Johns Hopkins School of Medicine, Baltimore, MD, USA; 5 Section for Biostatistics, Department of Public Health, Aarhus University, Aarhus C, Denmark; 6 Center for Diabetes Research, Gentofte University Hospital. Hellerup, Denmark

## Abstract

Several studies link increasing body mass index (BMI) to cognitive decline both as a consequence of obesity *per se* and as a sequela of obesity-induced type 2 diabetes. Obese individuals are prone to a chronic low-grade inflammation as the metabolically active visceral fat produces proinflammatory cytokines. Animal studies indicate that these cytokines can cross the blood–brain barrier. Such crossover could potentially affect the immune system in the brain by inducing gene expression of proinflammatory genes. The relationship between obesity and neuroinflammation in the human brain is currently unknown. Therefore we aim to examine the relationship between BMI and gene expression of central inflammatory markers in the human frontal cortex. Microarray data of 141 neurologically and psychiatrically healthy individuals were obtained through the BrainCloud database. A simple linear regression analysis was performed with BMI as variable on data on *IL10, IL1β, IL6, PTGS2* (COX2) and *NOS2* (iNOS). Increasing BMI is associated with a decrease in the mRNA expression of *IL10* (*P*=0.014) and an increase in the expression of *NOS2* (iNOS*; P*=0.040). Expressions of *IL10* and *NOS2* (iNOS) were negatively correlated (*P*<0.001). The expression of *IL10* was mostly affected by individuals with BMI ⩾40. Multiple linear regression analyses with BMI, age, sex and race as variables were performed in order to identify potential confounders. In conclusion, increasing BMI could affect the IL10-mediated anti-inflammatory defense in the brain and induce iNOS-mediated inflammatory activity.

## Introduction

The World Health Organization (WHO) estimates that about 1.9 billion adults are currently overweight,^1^ and obesity represents a massive economic burden on health-care systems worldwide. Much evidence links obesity in midlife to increased risk of dementia in later life.^2, 3, 4, 5, 6, 7^ However, a recent large-scale retrospective study of 2 million individuals reported that midlife obesity associates with a lower risk of dementia.^8^ To what extent obesity should be considered an independent risk factor for dementia remains to be settled. A better understanding of the link between obesity and neurodegeneration would be beneficial in the search for new therapeutic targets for common neurodegenerative diseases with an inflammatory component, such as Alzheimer's disease.

Several factors could contribute to an increased risk of developing dementia in the obese. Obese individuals have a higher prevalence of atherosclerosis,^9^ endothelial dysfunction and cerebral hypoperfusion. These are among the possible mechanisms of obesity-associated cognitive decline.^10, 11^ Obese individuals are more likely to develop diabetes, insulin resistance and/or metabolic syndrome (MetS). MetS is characterized by elevated plasma glucose levels, hypertension and dyslipidemia.^12^ It is estimated that 20–25% of all adults suffer from MetS and about 1 in 11 adults have diabetes, of which 90% have type 2 diabetes.^13, 14^ Type 2 diabetes patients carry a two to five times increased risk of both Alzheimer's disease and vascular dementia^15^ and MetS is a known risk factor for cognitive decline and overall dementia risk.^16^ Although the literature is not conclusive on the role of MetS in Alzheimer's disease development,^16^ the severity of Alzheimer's disease is greater in patients with MetS.^17^ Likewise, the role of insulin resistance in the brain is currently being investigated.^18^

Insulin resistance is linked to inflammation.^19^ Chronic systemic low-grade inflammation is a cardinal feature in obesity as visceral adipose tissue is a highly metabolically active organ that contributes to an increased level of proinflammatory cytokines such as interleukin (IL)-1β and IL6.^20, 21, 22^ Rodent studies have shown that circulating proinflammatory cytokines can cross the blood–brain barrier.^23, 24^ The communication between the brain and the periphery occurs via several routes.

Saturable carrier-mediated transport systems have been identified, which transport cytokines IL-1β, IL6 and tumor necrosis factor-α from the blood to the central nervous system (CNS).^23, 24^ Inflammatory cytokines interact with the circumventricular organs and the brain endothelium^24, 25^ and circulating proinflammatory cytokines are believed to activate perivascular macrophages and microglia, and also signal through receptors on the cerebral endothelial cells.^26, 27^ Systemic inflammation in rats triggers microglia and astrocytes to induce IL10, tumor necrosis factor-α, IL-1β and IL6 in cerebral cortex.^27^ Such activation can affect microglia function within the brain, and microglia activity has been proposed as the link between inflammatory stimuli and altered neuroplasticity.^28, 29, 30^

Knowledge about the relationship between obesity and inflammation in the human brain is relatively sparse, but obesity has been associated with decreased human white matter integrity.^31^ In rodents, on the other hand, several studies have shown that obesity and high-fat diets leads to increased gene or protein expression of inflammatory cytokines in the hypothalamus, neocortex and hippocampus.^19, 32, 33, 34, 35, 36, 37^

The aim of the present study was to examine the hypothesis that obesity *per se* will induce an inflammatory response in the human brain. Utilizing microarray data from the BrainCloud database (http://braincloud.jhmi.edu/) of the Lieber Institute for Brain Development, this study analyzes microarray data from frontal cortex in individuals without neurological and psychiatric diseases at the time of death. The potential effect of body mass index (BMI) on the gene expression of selected genes, that is, *IL10, IL6, IL1β*, *NOS2* (iNOS) and *PTGS2* (COX2) was investigated by performing simple linear regression analyses treating BMI as the continuous variable including all adult individuals (age⩾18 years) in the cohort (*n*=141). In order to describe the impact of morbidly obese individuals, additional simple linear regression analyses were performed (*n*=122) excluding all individuals with a BMI ⩾40 from the analyses.

Several studies indicate that increased inflammatory levels are part of the aging process in the brain.^38, 39, 40^ In order to investigate possible confounders, we performed multiple linear regression analyses with BMI, age, sex and race as explanatory variables.

## Materials and methods

### Demographics

The BrainCloud database (http://braincloud.jhmi.edu/) contains a collection of microarray data on post-mortem samples from the human frontal cortex (Brodmann’s area 9 and 46). The samples were collected from individuals aged 0–78 years. The BrainCloud cohort only includes neurologically and psychiatrically healthy individuals. Additional information on sex, race and BMI (defined as: weight (mass_kg_/height_m_^2^) at the time of death was also available. We excluded individuals <18 years of age (*n*=34), individuals with known diabetes (*n*=4) and individuals of whom no information on BMI was available (*n*=17) leaving a total of 141 samples (77 African-Americans, 56 Caucasians, 4 Asian and 4 Hispanic individuals) to be included in the analyses. A detail description of the demographics can be seen in Table 1. All tissue collection was performed with informed consent obtained from the next of kin. All the data were subsequently anonymized in accordance with the rules and regulations of the National Institute of Health (using protocol 90-M-0142).

### Genes

Gene expressions analyzed in this study are mRNA expression data. The data were obtained through the use of a complementary DNA microarray chip performed at the NIH/NHGRI microarray core facility using the Illumina Oligoset HEEBO7 chip. A detailed description of tissue preparation and data analysis of BrainCloud is available in Colantuoni *et al.*^41^

The genes *Il10, 1L1β, IL6, PTGS2* (COX2) and *NOS2* (iNOS) were selected for the analyses.

### Statistics

Simple linear regression analyses of the expression for each gene was performed treating BMI as a continuous exploratory variable. To investigate the impact of very high BMI, this analysis was also performed on all individuals with a BMI below 40. This was choosen because of the WHO classification of morbidly obesity (BMI ⩾40).^42^ To identify potential confounders, we performed multiple linear regression analyses including BMI, age, sex and race. This was done for each gene (each probe of each gene if more than one probe was available) treating BMI and age as continuous variables and race and sex as categorical variables. For each data set assumptions of the multiple linear regression model was examined. To this end, normal distribution of the residuals was investigated by inspecting a QQ plot, whereas the linearity and the homoscedasticity of residuals were assessed by inspecting a plot of the residuals against the explanatory variables. Moreover, a squared residual versus leverage plot was made in order to examine the impact of each single observation of the model. No outliers were removed from the analyses. To obtain data fulfilling the assumptions of the multiple regression model, mathematical transformation of some gene expression/BMI data sets was performed, that is, transformation of the gene expression data by an exponential function or the application of a natural logarithm transformation of the variable BMI. In all analyses, a level of 0.05 was considered statistically significant. All analyses were carried out using STATA version 12.1 (College Station, TX, USA).

## Results

**Figure 1:**
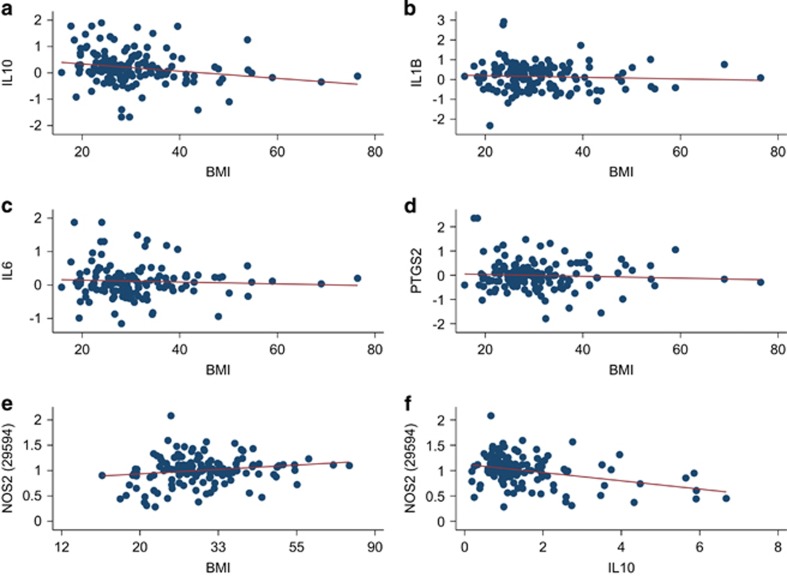
The figure shows simple linear regression models with mRNA expression level of *IL10, IL1β, IL6, PTGS2* and *NOS2* probe 29594 in relationship to increasing BMI (*n*=141; **a**–**e**). See Table 2a for details. The *y* axis presents arbitrary mRNA expression values and the *x* axis presents values of BMI. For *NOS2* probe 29594 the *y* axis presents arbitrary mRNA expression values transformed to an exponential function and the *x* axis presents values of natural logarithm transformed values of BMI. The figure also shows a simple linear regression model describing the relationship between mRNA expression levels of *IL10* in relationship to the mRNA expression levels of *NOS2* (iNOS; probe 29594*; n*=141). (**f**) For this model the *y* axis presents arbitrary mRNA expression values transformed to an exponential function and the *x* axis presents values of mRNA expression of IL10 transformed to an exponential function. (**a**) The mRNA expression level of *IL10* in relationship to increasing BMI (*P*=0.014). (**b**) The mRNA expression level of *IL1β* in relationship to increasing BMI (*P*=0.485). (**c**) The mRNA expression level of *IL6* in relationship to increasing BMI (*P*=0.518). (**d**) The mRNA expression level of *PTGS2* (COX2) in relationship to increasing BMI (*P*=0.468). (**e**) The mRNA expression level of *NOS2* (probe 29594) in relationship to increasing BMI (*P*=0.040). (**f**) The mRNA expression level of *IL10* in relationship to the mRNA expression of *NOS2* (iNOS; probe 29594), coefficient −0.0819 (−0.1190; −0.0447), F(1,139)=19.00, *R*^2^=0.120, *P*<0.001). BMI, body mass index; IL, interleukin.

Careful analysis of assumptions and squared residual versus leverage plots confirmed that the data sets could be appropriately analyzed applying a simple linear regression model and a multiple linear regression model. The relationship between BMI and the mRNA expression of the investigated inflammatory cytokines was not affected by age, sex and race. See Table 2a and b and Figure 1a–e for the simple linear regression analyses, and see Table 3 for the multiple linear regression analyses.

### BMI is associated with an altered mRNA expression of IL10 and iNOS, whereas BMI does not significantly affect the expression level of IL1β, IL6 and PTGS2 (COX2)

Performing a simple linear regression analysis, we found a significantly reduced *IL10* expression *P*=0.014 with increasing BMI (Figure 1a and Table 2a). The expression of *NOS2*, that is, *iNOS* (probe 29 594), was significantly upregulated with increasing BMI (*P*=0.040), whereas no significant effects of BMI were seen on *NOS2* probes 30 645 and 37 928 (*P*=0.136, *P*=0.801, respectively). The mRNA expression of the proinflammatory cytokines *IL1β*, *IL6* and *PTGS2* gene (COX2) was not significantly affected by increasing BMI (*P*=0.485, *P*=0.518 and *P*=0.468, respectively; Figure 1b–e and Table 2a).

In addition, we performed multiple linear regression models including age, race and sex in the analyses confirming the overall association between BMI and *IL10* and *NOS2* (iNOS) expression (Table 3). There was no statically significant effect of age, race and sex on the mRNA expression of *IL10*, *IL1β*, *IL6*, *PTGS2* and *NOS2* probe 29 594 and probe 37 928 (Table 3).

To examine the impact of the most markedly obese individuals (BMI ⩾40), we applied a simple linear regression analysis for BMI, omitting these individuals with BMI ⩾40 from the analyses. Looking at the remaining 122 individuals, the expression of *NOS2* remains significantly increased with increasing BMI for probes 29 594 and 30 645 (*P*=0.006 and *P*=0.006, respectively), whereas probe 37 928 is not statistically significantly affected (*P*=0.660; Table 2b). On the other hand, the statistical significance of the BMI induced alterations in the expression of *IL10* seemed to depend more on the highly obese individuals (*P*=0.258, simple linear regression analysis with *n*=122). As seen in Table 2b, the mRNA expression patterns of *IL1β*, *IL6* and *PTGS2* (COX2) were not significantly different when looking only at individuals with a BMI below 40.

### The mRNA expression of IL10 is inversely correlated with the expression of NOS2

Performing a simple linear regression analysis, we found that there was an inverse relationship between *IL10* and *NOS2* mRNA expression, assessing *NOS2* with probe 29 594 (*P*<0.001; Figure 1f). We found a similar significant inverse relationship between *IL10* and *NOS2* expression with *NOS2* probe 30 645 and *NOS2* probe 37 928 (*P*=0.017 and *P*=0.004, respectively).

### Increasing age is reflected by a significant downregulation of NOS2 (iNOS), whereas there is no significant effect of aging on PTGS2 (COX2), IL6, IL1β and IL10

In the multiple linear regression analyses, increasing age was associated with a significant downregulation of *NOS2* (probe 30645) (*P*=0.047; Table 3), whereas the expression of *NOS2* probe 29 594 and probe 37 928 was not significantly affected by increasing age (*P*=0.981, *P*=0.213, respectively). Aging had no significant effect of the expression level of *IL10, IL1β*, *IL6* and *PTGS2* (COX2) (*P*=0.205, *P*=0.996, *P*=0.467, *P*=0.285, respectively) in this cohort.

## Discussion

This study demonstrates that in prefrontal cortex of neurologically and psychiatrically healthy humans, a gradual increase in BMI is associated with discrete signs of altered gene expression, that is, reduced mRNA expression of the anti-inflammatory cytokine *IL10* and increased mRNA expression of *NOS2* (iNOS), albeit with a marked effect of the ~15% (*n*=19) morbidly obese individuals on the BMI-related changes in *IL10* expression. To the best of our knowledge, this study is the first to investigate the relationship between BMI and inflammatory gene expression in human brains without any neurological disease.

Accumulated evidence from animal studies suggests that active inflammation is a neuronal stress factor, which may *per se* affect higher mental functions such as cognition.^26, 30^ Increased levels of IL-1β and other inflammatory factors in CNS may damage synaptic function and inhibit long-term potentiation.^30^ Experimental studies of the endotoxin lipopolysaccharide in rodents support the notion that activation of microglia partly occurs through the toll-like receptor 4 (TLR4), subsequently resulting in the decrease in long-term potentiation.^29, 43^

Unlike the rodent studies showing a notable increase in IL-1β within brain tissue,^33, 34, 36^ mRNA expression of the proinflammatory genes *IL1β*, *IL6* and *PTGS2* (COX2) appeared to be unrelated to BMI in our study sample. These differences might reflect that this study focused on a neocortical area rather than hypothalamic areas, which are outside the limitations of the blood–brain barrier. One study does describe an association between inflammation in cortical and hippocampal regions and BMI.^34^ Given the sample size, we cannot be certain that additional inflammatory features could be present selectively in extremely obese individuals, that is, BMI> 40–45, but the present study sample included only 19 morbidly obese individuals limiting our analyses. Supporting an effect of severe obesity, the association between BMI and *IL10* expression was only significantly affected when analyses included the 19 individuals with a BMI ⩾40. Expression of *NOS2* was still significantly upregulated when excluding the most obese individuals, although expression of probe 37 928 designed to fit an alternative isoform of the *NOS2* gene was not statistically affected by BMI. Both the importance of *NOS2* isoforms for the activity of this gene and the actual number of individuals displaying this alternative splice variant in our cohort is unknown—but our findings might simply reflect that only few individuals display the alternative isoform detected by probe 37 928. Perhaps more surprisingly, the probe 30 645, which targets a constitutive portion of the *NOS2* gene and displays some overlap with the location of the 29 594 probe, appears unaffected by increasing BMI when including all 141 individuals. However, looking at the coefficients for the BMI impact on gene expression in Table 2a it appears that the effect of BMI on *NOS2* expression is similar for the two groups and a significant increase with BMI is seen in both cases when excluding the 19 morbidly obese individuals (Table 2b). With the heterogeneity of our sample—in which variations in, eating habits, D-vitamin status and so on are likely to be present—it is noteworthy that the mRNA expressions of both *IL10* and *NOS2* (iNOS) display an association with increasing BMI in this relatively small cohort. Still, this emphasizes the need for further studies supporting the present microarray findings, through deep RNA sequencing, qPCR and/or protein expression.

Lending support to a potential biological relevance of the altered mRNA expression of *NOS2* (iNOS) in the present study, we saw a negative relationship between the increased mRNA expression of *NOS2* and the reduced mRNA expression of *IL10* (Figure 1f). An attenuated microglial production of nitric oxide as a response of microglia cultured with IL10 has been reported in rats.^44^ IL10 also downregulates mRNA expression of *iNOS* in human macrophages.^45^ Moreover, IL10-deficient mice injected with lipopolysaccharide respond with a higher expression of iNOS than their wild-type counterparts.^46^ Despite the production of IL10 in adipose tissue^22^ others have found a reduced IL10 level in the blood of obese individuals.^47^ On the other hand, Esposito *et al.*^48^ found a higher IL10 level in the blood of obese individuals compared with their lean counterparts, but also identified a subgroup of both obese and non-obese individuals suffering from MetS who had significantly lower circulating IL10 levels.

Local production of IL10 in CNS promotes neuronal and glial survival.^49^ Reduced IL10 levels in the brain would likely increase the sensitivity of the brain toward harmful stimuli. In a murine study, IL10 in the subventricular zone modulates ERK and STAT3 activity. Via these factors, IL10 may play a role in adult neurogenesis,^50, 51^ hence linking Il10 levels to cognitive abilities. Although the role of obesity as such in cognitive performance is not clear-cut,^52^ several studies found a negative effect of obesity on cognition, leading to mild cognitive impairment.^53, 54^ Moreover, there are beneficial effects of weight loss on brain function, such as improved verbal memory, executive functions and global cognition, have been reported in mild cognitive impairment patients.^53, 54^

In the present study we applied simple linear regression analyses to evaluate the effects of BMI on gene expression. We used multiple linear regression analyses to elucidate whether potential confounders such as age, sex and race affected the results. Similar effects of BMI were seen in both analyses. The resulting *R*^2^ values in both the simple linear and the multiple linear regression analyses are, however, while statistically significant, relatively small. This is likely influenced by the nature of the sample in which the number of individuals with very high BMI was somewhat smaller than the large number with average BMI. We believe our findings in conjunction with recent literature point toward a need for further studies of both the impact of BMI and the role of IL10 in the brain during metabolic and inflammatory challenges.

When setting up the study, we included the effect of aging on the investigated genes; however, little impact of age was seen despite the apparent small but significant downregulation of *NOS2* on the 37 928 probe. In a previous study using the same data set, others have found a correlation between age and alterations in expression levels of *NFKB1, TRAF6, TLR4, IL1R1, BDNF* and *NGF* among others. However, they have not investigated the role of BMI.^55^ Others have described increased microglia activation in the human brain with increasing age^56^ and an overall increase in inflammatory activity, which might become detrimental in old age.^57^ Our cohort is relatively young, and we cannot conclude that neuroinflammation will not be a problem in senescent.

In conclusion, in a population of 141 non-diabetic adult individuals with no known psychiatric or neurological disease, we have found an association between altered gene expression in prefrontal cortex and increasing BMI levels involving a decreased mRNA expression of *IL10* and an increased mRNA expression of *NOS2* (iNOS) despite indication of an age-related downregulation of this gene in our population. In light of the increasing prevalence of obesity, further research into the long-term effects of obesity on the brain is needed to obtain a better understanding of the underlying mechanisms linking obesity, aging and brain inflammation.

## Figures and Tables

**Table 1:** Demographics of the cohort divided according to the international WHO classification 57 of adult underweight, normal weight, overweight, obesity and morbidly obesity

*Demographic of the cohort divided according to WHO BMI classification*					
	*BMI*	n *(141)*	*Sex*	*Race*	*Mean age*
Underweight	<18.5	3	3M	3AA	45.0
Normal weight	18.5–24.99	33	15M/18F	15AA/17C/1A	44.2
Overweight	25–29.99	44	38M/6F	19AA/21C/3A/1H	45.5
Obesity	30–39.99	42	29M/13F	23AA/17C/2H	43.8
Morbidly obese	⩾40	19	8M/11F	17AA/1C/1H	41.9

Abbreviations: A, Asian; AA, African-American; C, Caucasian; BMI, body mass index; F, Female; H, Hispanic; M, Male; WHO, World Health Organization. Additional information of the cohort. Mean BMI of the cohort: 30.8 (M: 29.9, F: 32.7), mean age of the cohort: 44.2 (M: 42.3, F: 48.3), the M/F ratio: 96M/45F and race: 77AA/56C/4A/4H.

Underweight: BMI <18.5, normal weight: BMI 18.5–24.99, overweight: BMI 25–29.99, obesity: BMI 30–39.99 and morbidly obesity (obese class 3): BMI ⩾40.

**Table 2:** Summary of findings from the simple linear regression models with BMI as factor

*a. Simple linear regression models with* n=*141*									
*Gene*	*Probe number*	*Probe type*	*Factor*	*Coeff.*	*CI interval*	*s.e.*	R*^2^*	*F(1,139)*	P*-value*
*IL10*	8663	hHC	BMI	−0.0137	(−0.0245; −0.0028)	0.005	0.0425	6.17	0.014
*IL1B*	22 194	hHC	BMI	−0.0041	(−0.0158; 0.0075)	0.006	0.0035	0.49	0.485
*IL6*	36 684	hHA	BMI	−0.0028	(−0.0112; 0.0056)	0.004	0.0030	0.42	0.518
*PTGS2 (COX2)*	13 444	hHC	BMI	−0.0038	(−0.0142; 0.0065)	0.005	0.0038	0.53	0.468
*NOS2 (iNOS)*	29 594	hHR	BMI	0.1733	(0.0077; 0.3389)	0.084	0.0299	4.28	0.040
*NOS2 (iNOS)*	30 645	hHC	BMI	0.2259	(−0.0718; 0.5235)	0.151	0.0159	2.25	0.136
*NOS2 (iNOS)*	37 928	hHA	BMI	−0.0005	(−0.0048; 0.0037)	0.002	0.0005	0.06	0.801

*b. Simple linear regression models with* n=*122 (individuals with BMI>40 are excluded)*									
*Gene*	*Probe number*	*Probe type*	*Factor*	*Coeff.*	*CI interval*	*s.e.*	R*^2^*	*F(1,120)*	P*-value*
*IL10*	8663	hHC	BMI	−0.0124	(−0.0339; 0.0092)	0.011	0.0106	1.29	0.258
*IL1B*	22 194	hHC	BMI	−0.0015	(−0.0246; 0.0217)	0.012	0.0001	0.02	0.900
*IL6*	36 684	hHA	BMI	−0.0039	(−0.0211; 0.0132)	0.246	0.0017	0.20	0.652
*PTGS2 (COX2)*	13 444	hHC	BMI	−0.0148	(−0.0348; 0.0052)	0.010	0.0175	2.14	0.146
*NOS2 (iNOS)*	29 594	hHR	BMI	0.3610	(0.1045; 0.6175)	0.130	0.0608	7.77	0.006
*NOS2 (iNOS)*	30 645	hHC	BMI	0.6578	(0.1928; 1.1228)	0.235	0.0614	7.84	0.006
*NOS2 (iNOS)*	37 928	hHA	BMI	−0.0019	(−0.0104; 0.0066)	0.004	0.0016	0.19	0.660

Abbreviations: BMI, body mass index; CI, confidence interval; coeff., coefficient; hHA, human alternative exonic; hHC, human constitutive exonic; hHR, human mRNA; IL, interleukin.

(a) Simple linear regression models with *n*=141. (b) Simple linear regression models with *n*=122 (excluding individuals (*n*=19) with BMI ⩾40 from the analyses).

From the left: gene (the name of the investigated gene); probe number (the number identifying the probe in http://braincloud.jhmi.edu/); probe type; factor (the parameter BMI in simple the linear regression model); coefficient (the arbitrary slope value); *R*^2^ of the model; F-value; *P*-value.

**Table 3:** Summary of findings from the multiple linear regression models (*n*=141) with BMI, age, sex and race as factors

*Multiple linear regression models with n*=*141*									
*Gene*	*Probe number*	*Probe type*	*Factors*	*Coeff.*	*CI interval*	*s.e.*	R*^2^*	*F(6,134)*	P*-value*
*IL10*	8663	hHC	BMI	−0.0131	(−0.0247; −0.0015)	0.006	0.064	1.53	0.027
			Age	−0.0050	(−0.0128; 0.0028)	0.004			0.205
			Sex	0.0019	(−0.2345; 0.2382)	0.119			0.988
			Race	−0.0887	(−0.3205; 0.1431)	0.117			0.451
*IL1B*	22 194	hHC	BMI	−0.0046	(−0.0169; 0.0077)	0.006	0.039	0.91	0.463
			Age	−0.00002	(−0.0083; 0.0083)	0.004			0.996
			Sex	0.1190	(−0.1326; 0.3706)	0.127			0.351
			Race	0.1715	(−0.0752; 0.4183)	0.125			0.171
*IL6*	36 684	hHA	BMI	−0.0060	(−0.0149; 0.0029)	0.004	0.042	0.98	0.184
			Age	−0.0022	(−0.0082; 0.0038)	0.003			0.467
			Sex	−0.1145	(−0.2955; 0.0666)	0.092			0.213
			Race	0.1379	(−0.0397; 0.3155)	0.090			0.127
*PTGS2 (COX2)*	13 444	hHC	BMI	−0.0040	(−0.0149; 0.0070)	0.006	0.035	0.82	0.475
			Age	−0.0040	(−0.0114; 0.0034)	0.004			0.285
			Sex	0.1369	(−0.0870; 0.3608)	0.113			0.229
			Race	−0.0130	(−0.2326; 0.2066)	0.111			0.907
*NOS2 (iNOS)*	29 594	hHR	BMI	0.2012	(0.0253; 0.3771)	0.089	0.044	1.03	0.025
			Age	−0.00004	(−0.0035; 0.0034)	0.002			0.981
			Sex	0.0479	(−0.0558; 0.1517)	0.052			0.362
			Race	−0.0252	(−0.1266; 0.0762)	0.051			0.624
*NOS2 (iNOS)*	30 645	hHC	BMI	0.2543	(−0.0535; 0.5621)	0.156	0.081	1.96	0.105
			Age	−0.0061	(−0.0121; −0.00009)	0.003			0.047
			Sex	0.0946	(−0.0869; 0.2761)	0.092			0.305
			Race	−0.1334	(−0.3109; 0.0440)	0.090			0.139
*NOS2 (iNOS)*	37 928	hHA	BMI	−0.0004	(−0.0049; 0.0041)	0.002	0.067	1.59	0.859
			Age	0.0019	(−0.0011; 0.0049)	0.002			0.213
			Sex	−0.0854	(−0.1765; 0.0056)	0.046			0.066
			Race	−0.0197	(−0.1090; 0.0696)	0.045			0.663

Abbreviations: BMI, body mass index; CI, confidence interval; coeff., coefficient; hHA, human alternative exonic; hHC, human constitutive exonic; hHR, human mRNA; IL, interleukin.

From the left: Gene (the name of the investigated gene); probe number (the number identifying the probe in http://braincloud.jhmi.edu/); probe type; factors (the parameters BMI, age, sex and race in the multiple regression model); coefficient (the arbitrary slope value for BMI and age; for sex it means the difference in expression between males and females; for race it means the difference in expression between African-Americans and Caucasians); *R*^2^ of the model; F-value; *P*-value.
